# A High-Throughput, Flow Cytometry-Based Method to Quantify DNA-End Resection in Mammalian Cells

**DOI:** 10.1002/cyto.a.22155

**Published:** 2012-08-14

**Authors:** Josep V Forment, Rachael V Walker, Stephen P Jackson

**Affiliations:** 1 The Gurdon Institute and Department of Biochemistry, University of CambridgeCB2 1QN Cambridge, United Kingdom; 2 Cambridge Stem Cell Institute, Flow Cytometry Core Facility, Wellcome Trust Centre for Stem Cell Research, University of CambridgeCB2 1QR Cambridge, United Kingdom

**Keywords:** flow cytometry, replication protein A, DNA damage, DNA replication, DNA repair, homologous recombination, DNA-end resection

## Abstract

Replication protein A (RPA) is an essential trimeric protein complex that binds to single-stranded DNA (ssDNA) in eukaryotic cells and is involved in various aspects of cellular DNA metabolism, including replication and repair. Although RPA is ubiquitously expressed throughout the cell cycle, it localizes to DNA replication forks during S phase, and is recruited to sites of DNA damage when regions of ssDNA are exposed. During DNA double-strand break (DSB) repair by homologous recombination (HR), RPA recruitment to DNA damage sites depends on a process termed DNA-end resection. Consequently, RPA recruitment to sub-nuclear regions bearing DSBs has been used as readout for resection and for ongoing HR. Quantification of RPA recruitment by immunofluorescence-based microscopy techniques is time consuming and requires extensive image analysis of relatively small populations of cells. Here, we present a high-throughput flow-cytometry method that allows the use of RPA staining to measure cell proliferation and DNA-damage repair by HR in an unprecedented, unbiased and quantitative manner. © 2012 International Society for Advancement of Cytometry

In response to DNA damage, cells elicit signal transduction cascades, globally termed the DNA-damage response (DDR), whose primary aim is to promote effective DNA repair (1,2). Key features of the DDR are activation of DNA repair pathways and the triggering of DNA-damage checkpoints that delay cell-cycle progression and allow more time for repair. If repair is unsuccessful or the amount of DNA damage is excessive, cells generally either undergo apoptosis or enter into a permanent cell-cycle withdrawal state known as senescence. Not surprisingly, DDR defects are associated with genomic instability and tumor development, and hereditary mutations leading to DDR impairment cause heightened cancer predisposition as well as various other age-related pathologies [reviewed in (1,2)].

Of the wide range of lesions that DNA can receive, the most cytotoxic appears to be the DNA double-strand break (DSB). Because only one or a few unrepaired DSBs can result in cell death, life has evolved systems to efficiently detect DSBs and mediate their repair (3). Two main, mutually exclusive DSB repair pathways exist. The first of these, non-homologous end joining (NHEJ), operates throughout the cell cycle and, through the coordinated actions of various proteins, leads to the stitching together of the broken double-stranded DNA ends. Although it can result in faithful repair, NHEJ is regarded as an error-prone mechanism because it often leads to clustered mutations at the repair site (4). By contrast, homologous recombination (HR) generally only operates during S and G2 phases of the cell cycle and, given that it uses the sister chromatid as a template for DSB repair, it is usually an error-free mechanism (5). Recent work has indicated that HR defects exist in various cancer cells, with inherited mutations in HR-promoting factors such as BRCA1 and BRCA2 leading to elevated cancer predisposition (2,6).

One of the first events taking place at a DSB being repaired by HR is the 5′ to 3′ nucleolytic degradation of one DNA strand, a phenomenon termed DNA-end resection (7). The region of single-stranded DNA (ssDNA) generated during this process is then rapidly covered by replication protein A (RPA), a stable protein complex formed by three subunits: RPA70/RPA1, RPA32/RPA2 and RPA14/RPA3. RPA binding is crucial for stabilization of resected DSBs and for them to subsequently initiate the strand-invasion steps of HR that are promoted by various factors, including BRCA2 and RAD51 (7). Notably, RPA also binds to and stabilizes ssDNA regions formed at the DNA replication fork, and is thus essential for cell proliferation (8). In addition, RPA-ssDNA binds to the ATRIP protein in association with the protein kinase ATR, whose ensuing activation at sites of resected DNA then triggers DNA-damage checkpoint signaling as well as more directly promoting DNA repair and replisome stability (9)**.** In light of the above issues, defects in resection are associated with defects in both HR and DNA-damage signaling.

To complement assays that assess HR by measuring its end points (10–13), and as a rapid means of detecting proteins involved in HR-mediated repair, many researchers have used microscopic detection of RPA or other HR-associated proteins within punctate subnuclear foci at DNA damage sites to assess progression into and through the various stages of HR (14–18). Although defects in RPA-focus formation as measured by these methods can be clear when core resection proteins are mutated or absent, recent research has highlighted the fact that many proteins play a role in this process (19), with defects in some of them leading to often quite subtle phenotypes that can be difficult to quantify by standard microscopy-based assays. As described below, to help circumvent such shortcomings, we have developed a flow-cytometry method to quantitatively analyze RPA accumulation as readout for DNA-end resection, a method that should find utility in various studies analyzing HR and associated events and in defining how various proteins influence such processes.

## MATERIALS AND METHODS

This report is presented in a manner that is fully compliant with the Minimum Information about a Flow Cytometry Experiment (MIFlowCyt) standard.

### Cell Lines, Reagents, and Transfection

Human osteosarcoma U2OS cells (ATCC #HTB-96) were used throughout and were grown in Dulbecco's modified Eagle medium supplemented with 10% fetal bovine serum, glutamine, and antibiotics. Camptothecin and etoposide were from Sigma. Transfection with small-interfering RNAs (siRNAs) was performed by using Lipofectamine RNAiMAX (Invitrogen) following the manufacturer's instructions. siRNA sequences used were described previously (15). All experiments were carried out between September 2011 and March 2012.

### Antibodies and Cell Cycle Reagents

Primary antibodies: mouse anti-RPA32 (RPA2 Ab #1; RPA34-20 Merck NA19L, 1:100 dilution), mouse anti-RPA32 (RPA2 Ab #2; RPA2 9H8 Abcam ab2175, 1:200 dilution), rabbit anti-γH2AX (Histone H2A.X phospho-Ser139, Cell Signaling 2577, 1:100 dilution). Secondary antibodies: goat anti-mouse Alexa Fluor 488 (Molecular probes, 1:200 dilution), goat anti-rabbit Alexa Fluor 647 (Molecular probes, 1:200 dilution). 5-ethynyl-2′-deoxyuridine (EdU) incorporation was measured by using the Click-iT EdU Alexa Fluor 647 Flow cytometry kit (Life Technologies) following manufacturer's instructions.

### Sample Preparation for Flow Cytometry

Samples were collected from 6-cm dishes, with cells being 60–90% confluent (∼ 0.5-1 x 10^6^ cells). If EdU detection was performed, cells were pulse-labeled with 10 μM EdU for 30 min before collection. Cells were harvested by trypsinization. After washing with 1x phosphate-buffered saline (PBS), cells were fixed and permeabilized with 100 μl of BD Cytofix/Cytoperm buffer (BD Biosciences) for 15 min at room temperature (alternatively, fixation can be done in 4% paraformaldehyde in 1xPBS for 15 min, and permeabilization in 1x PBS containing 0.2% Triton X-100 (PBS-T) for 30 min). For RPA2 staining, extraction of non-chromatin bound RPA2 was performed prior fixation by resuspending pelleted cells in 100 μl of PBS-T and incubating for 10 min on ice. After extraction cells were washed with 2 ml of 1x PBS containing 1 mg/ml of bovine serum albumin (PBS-BSA), and then fixed/permeabilized. After fixation/permeabilization cells were washed with 0.5 ml of 1x BD Perm/Wash buffer (BD Biosciences; alternatively with PBS-BSA) and resuspended in 50 μl of 1x BD Perm/Wash buffer with the appropriate dilution of primary antibodies. After at least 1 h incubation at room temperature, cells were washed with 0.5 ml of 1x BD Perm/Wash buffer. Cell pellets were then resuspended in 50 μl of 1x BD Perm/Wash buffer with the appropriate dilutions of secondary antibodies, and incubated for 30 min at room temperature in the dark. If dual antibody/EdU staining was performed, the EdU detection reaction was carried out at this point. After washing with 0.5 ml of 1x BD Perm/Wash buffer, cells were resuspended in 0.5 ml of 1x PBS containing 0.02% sodium azide, 250 μg/ml RNase A and 2 μg/ml of 4′,6-diamidino-2-phenylindole (DAPI), then incubated at 37°C for 30 min in the dark.

### Analysis of Flow Cytometry Samples

Samples were analyzed by using a Beckman Coulter CyAn ADP Flow Cytometer. DAPI was excited with a 405 nm laser and emissions collected via a 450/50 filter. The Alexa 488 fluorochrome was excited by a 488 nm laser, and the emitted light collected via a 530/40 filter. A 635 nm laser was used for the Alexa 647 fluorochrome and the emission collected via a 670/30 filter. As each of the fluorochromes was excited by a different laser no compensation was necessary. Cells were gated on the Forward versus Side Scatter plot to eliminate debris, and then single cells were gated by using a dot-plot showing the pulse height versus pulse area of the DAPI channel (see Supporting Information Fig. S1). Postacquisition analysis was performed with FlowJo software (Tree Star).

## RESULTS

### RPA Antibodies Can be Used to Assess Actively Replicating Cells

**Figure 1:**
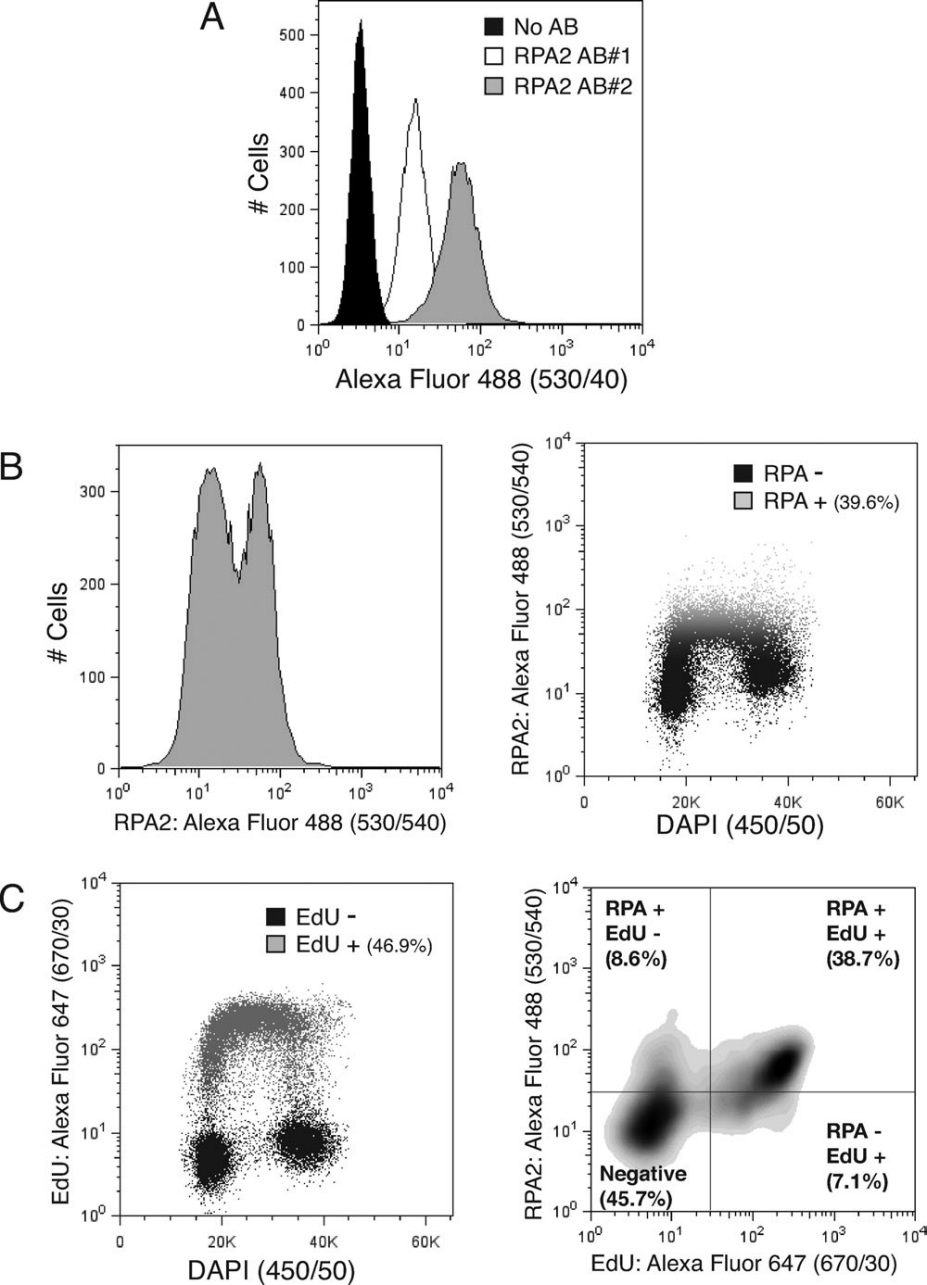
**A:** Anti-RPA2 antibodies can be used in flow cytometry. The *x* axis represents the intensity of RPA2 signals (logarithmic scale). In the control sample (No Ab) only the secondary antibody was used. **B:** Extraction of samples prior to fixation differentiates between two populations of cells with regards RPA2 staining (left panel). When RPA2 signals are compared with total DNA content (DAPI), the RPA2-positive cells correspond to those in S phase (right panel). **C:** Most cells that are RPA2 positive are also positive for EdU incorporation. Left panel: extent of EdU incorporation compared with DNA content (DAPI). Right panel: comparison of EdU incorporation and RPA2-positive cells. Gating in the right panel was established using the gating in the left panel (for EdU) and in the right panel in (B) (for RPA2). In A, 10,000 events were counted per condition. In the rest of panels, 30,000 events were counted per condition.

In order to detect the presence of the RPA complex in human osteosarcoma U2OS cells by flow cytometry, we initially used two different antibodies raised against the RPA2 subunit of the complex that have been validated in immunofluorescence experiments (15,20). Both antibodies gave reasonable intensity signals that warranted further use, although we decided to use one of these, RPA2 antibody #2, for subsequent experiments because it produced higher signal intensities (Fig. 1A and Materials and Methods section).

Although the RPA complex is ubiquitously expressed throughout the cell cycle, its binding to ssDNA is largely restricted to cells undergoing DNA replication (8). Unlike most nucleoplasmic proteins, factors tightly bound to chromatin and/or DNA tend to be resistant to extraction with detergents or increasing salt concentrations, characteristics that have been the basis for cellular fractionation (or “chromatin fractionation”) experiments (21,22). To assess whether we could distinguish between free and DNA-bound RPA by flow cytometry, we treated cells with detergent prior to fixation (see Materials and methods and Ref. 23). As shown in Figure 1B (left panel), extraction of soluble RPA2 before fixation resulted in the appearance of two different but overlapping cell populations with regards of RPA2 staining. Notably, when compared with total DNA content by staining with DAPI, the RPA-positive cell population appeared to represent cells in S phase (Fig. 1B, right panel). To more directly investigate this connection, we pulse-labeled cells with the nucleotide analogue EdU, extracted them and performed dual staining by using click chemistry to detect EdU (24) together with anti-RPA2 antibodies (see Materials and methods). Analyses of the resulting samples established that most cells staining positive for RPA were also EdU positive (Fig. 1C). Taken together, these results showed that RPA staining after extraction can be used in flow cytometry as a way to detect cells undergoing DNA replication.

### DNA Damage Causes Increased Intensity of RPA Signals

**Figure 2:**
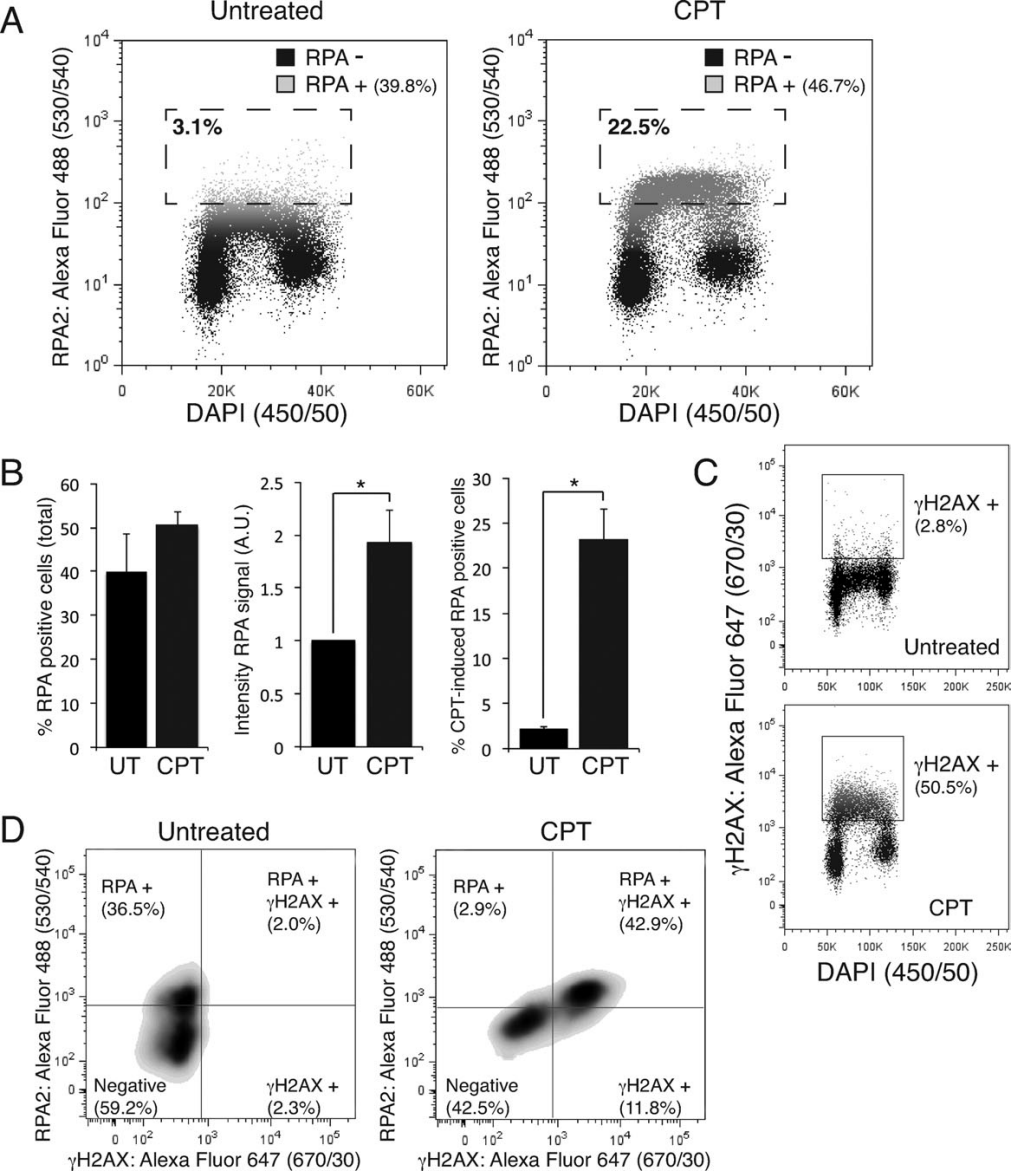
A: DNA damage increases the intensity of RPA2 signals. Cells were treated with 1 μM of camptothecin (CPT) for 1 h before harvesting. The dashed square marks the gate (showing the percentage of cells in it) used for quantification in the right panel in (B). **B:** Quantification of total amounts of RPA2-positive cells (left panel), the intensities of RPA2 signals (normalized to the intensity in untreated samples; middle panel), and the total amounts of DNA-damage induced RPA-positive cells (using the population gated in (A); right panel). For an example of the gating scheme, see Supporting Information Figure S1. Results are averages of at least three independent experiments and the error bars correspond to standard deviations. (*) Denotes statistically significant differences (*P* value < 0.05). **C:** DNA damage causes the appearance of γH2AX signals. Cells were treated as in (A). **D:** Comparison between RPA2 and γH2AX signals. Most RPA2-positive cells are also positive for γH2AX. In all panels 30,000 events were counted per condition.

Agents that cause DNA damage or DNA replication stress are known to produce local accumulation of RPA into focal structures that can be readily observed by immunofluorescence analyses of fixed cells (14). To test whether DNA damage could also change the pattern of RPA2 staining observed by flow cytometry, we treated U2OS cells with camptothecin (CPT), an inhibitor of DNA topoisomerase I (TopI) that causes the formation of TopI-DNA covalent adducts that are then converted to DSBs in S-phase when they are encountered by active replication forks (25). As shown in Figure 2A, when we analyzed cells by flow cytometry, CPT treatment led to a clear increase in RPA2 signal intensity within S-phase cells (for an example of the gating scheme, see Supporting Information Fig. S1). Quantification revealed that, while the overall proportion of cells exhibiting RPA2 staining did not significantly change upon CPT treatment (Fig. 2B, left panel), the intensity of RPA2 signal increased approximately 2-fold (Fig. 2B, middle panel; for an alternative way to measure differences in RPA2 staining see Supporting Information Fig. S2). To more clearly reflect the differences in RPA2 staining between untreated and treated cells, we defined a gate at the higher intensity level of RPA staining for most cells (>95%) in untreated conditions (dashed square in Fig. 2A; see Supporting Information Fig. S1) and used this as the basis for further quantifications. Strikingly, when this new gate was applied to define DNA-damage induced RPA positivity, the difference between untreated and CPT-treated samples was now very dramatic (Fig. 2B, right panel).

One of the earliest markers for DDR activation is phosphorylation of histone variant H2A.X on Ser-139 to yield the phosphorylated species termed γH2AX (6). Given that CPT treatment preferentially causes DNA damage in actively replicating cells (26), as might have been expected, our analyses mainly detected γH2AX signals in S-phase cells (Fig. 2C). To determine whether the increased intensity on RPA2 staining we observed after CPT treatment correlated with the appearance of γH2AX signals, we subjected extracted cells to dual labeling with anti-RPA2 and anti-γH2AX antibodies. As shown in Figure 2D, this established that the majority of RPA-positive cells after CPT treatment were also γH2AX positive. Collectively, these results confirm that our flow-cytometry based assay can readily detect increased intensity of RPA2 signals in cells harboring marks of DNA damage.

### RPA Staining Can Be Used as Readout of DNA-End Resection

**Figure 3:**
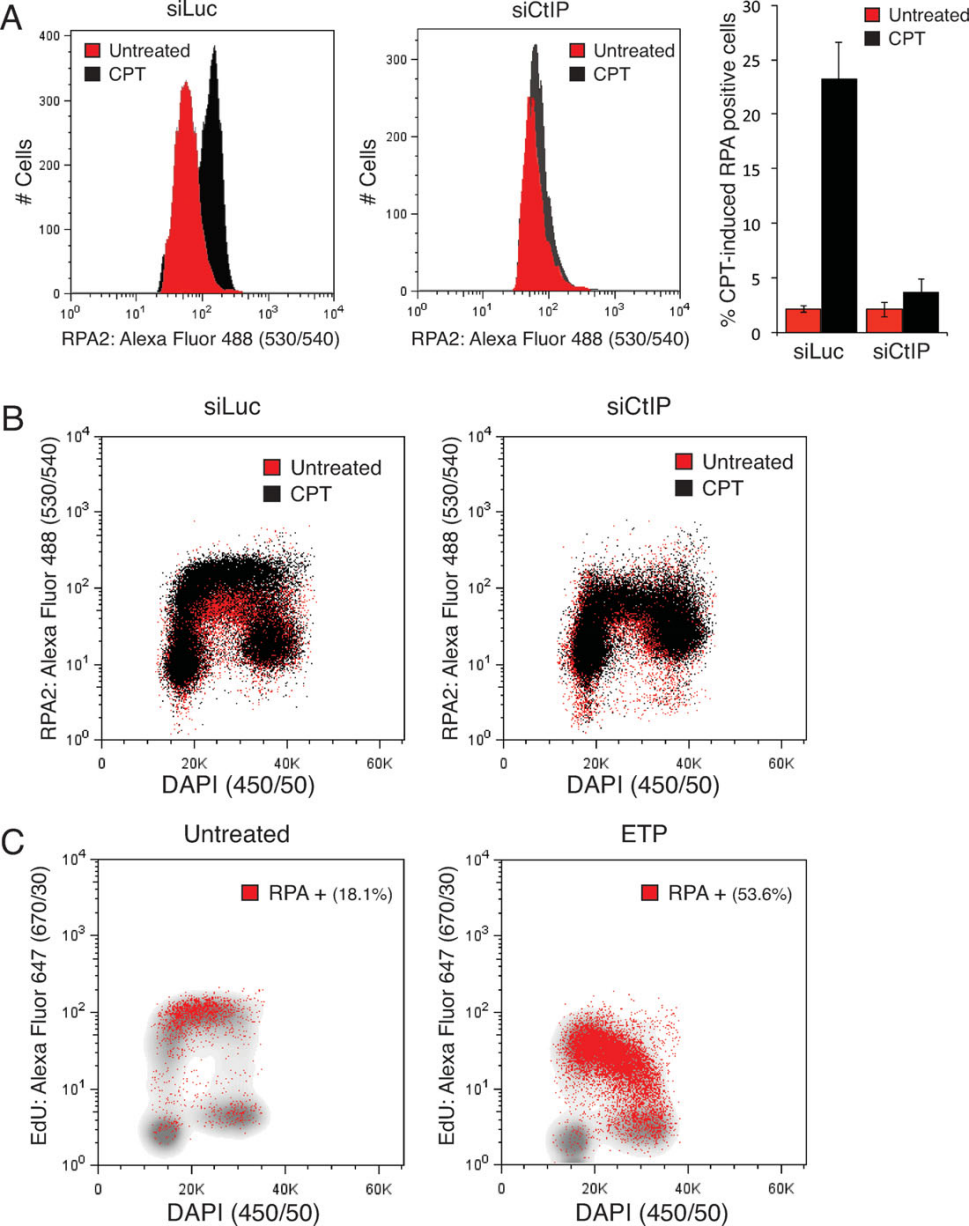
A: CPT-induced increase in RPA2 staining reflects DNA-end resection. Cells were transfected with control (luciferase, Luc) or CtIP siRNAs 48 h before the experiment. Cells were treated as in Figure 2A. Quantifications (right panel) were performed with the same gating scheme as in Figure 2B (see Supporting Information Fig. S1). Results are averages of at least three independent experiments and the error bars correspond to standard deviations. **B:** CtIP depletion only affects DNA-damage induced RPA2 staining. Cells were treated as in (A). **C:** Etoposide (ETP) increases the amount of RPA2-positive cells in S and G2 phases of the cell cycle. Cells were treated with 5 μM ETP for 4 h before harvesting. RPA2-positive populations (red dots) are plotted on top of the EdU incorporation profiles (in gray). In all panels 30,000 events were counted per condition. [Color figure can be viewed in the online issue, which is available at wileyonlinelibrary.com.]

When cells are treated with DNA-damaging agents, formation of regions of RPA-coated ssDNA that are detectable by immunofluorescence techniques primarily reflects two effects: uncoupling of the normally synchronized movements of replicative DNA polymerases and helicases at ongoing replication forks (27); and DNA-end resection at sites of DNA breaks. CPT causes DSBs in S phase that are resected by the concerted actions of several DNA helicases and nucleases in a process that is essential for DNA repair by HR (7). Key amongst these proteins is CtIP that, together with the MRE11-RAD50-NBS1 complex, plays a major role in initiating resection, with CtIP impairment causing a substantial defect in RPA-coated ssDNA accumulation after CPT treatment (15). To test whether we could detect this defect with our flow-cytometry based assay, we used siRNAs to deplete CtIP protein levels in human U2OS cells. When compared to the mock-depleted control, CtIP-depleted cells exhibited a dramatic decrease in the ability of CPT to heighten RPA positivity in cells (Fig. 3A). Importantly, and consistent with the known functions of CtIP, depletion of this factor strongly affected the CPT-induced increase of RPA staining but had no effect on normal RPA staining levels in the S phase population of cells (Fig. 3B).

In eukaryotic cells, DSB repair by HR requires the presence of an undamaged, homologous DNA molecule and, because this molecule is invariably the sister chromatid, this kind of DNA repair pathway is restricted to the S and G2 cell cycle phases. Accordingly, being a key control point for initiation of HR, DNA-end resection is also mainly only readily detectable in S and G2 cells (7). To test whether DNA-damage induced RPA staining can also be detected through our flow-cytometry based assays in G2 cells, we used etoposide (ETP), an inhibitor of DNA topoisomerase II (TopII) that yields TopII-DNA covalent adducts and produces reactive oxygen species. Due to the mechanism-of-action of TopII enzymes, collision of either DNA replication forks or transcription units with these DNA-protein complexes results in the formation of DNA DSBs in all cell-cycle stages (28,29). As shown in Figure 3C, U2OS cells treated with ETP exhibited accumulation of RPA2 staining not only in actively replicating cells (as measured by EdU incorporation), but also in G2 cells. By contrast, and in line with the known cell-cycle control mechanisms governing resection (see above), we observed no ETP-induced increase in RPA2 staining in G1 cells.

## DISCUSSION

We have developed a flow-cytometry based assay that uses anti-RPA antibodies to detect RPA-ssDNA formation in cells. By extracting non DNA-bound RPA before sample fixation, we were able to distinguish non-replicating from replicating cells, thus establishing RPA as a new marker for cellular proliferation that can be employed in flow cytometry studies. We have also shown that this assay can identify increased RPA signals caused by DNA-damaging agents. The fact that the assay can detect significant differences in signals after DNA damage in both the percentage of cells showing RPA staining and the intensity of RPA staining in a given cell population opens the possibility of its use as a more quantitative and unbiased way to measure RPA-coated ssDNA formation in various experimental settings.

Absence or dysfunction of proteins that affect RPA focus formation after DNA DSB formation usually results in defects in DNA repair by HR and can also impair DNA-damage signaling via ATR and its downstream targets, including the checkpoint kinase CHK1 (15,30). Because of this, there has been considerable effort expended by researchers in using RPA focus formation as measure of resection and HR competency. While successful, such approaches are subject to identification of false positives due to the fact that depleting some factors yields RPA focus formation defects, not because of impaired resection or RPA loading per se but because the depletion results in a reduction of the S-G2 population of cells in which DNA-end resection (and consequently HR) takes place (11). The assay method that we have developed largely circumvents this problem – thus helping in the identification of genuine effectors of DNA-end resection and HR – by allowing the simultaneous and quantitative measurement of both RPA accumulation on DNA together with measurement of cells displaying ongoing DNA replication through EdU incorporation. It is noteworthy that various proteins with sometimes overlapping roles have been implicated in the generation of RPA-coated ssDNA (19). We anticipate that the unbiased and high-throughput quantitative way we have developed to measure changes on RPA accumulation will help investigators to establish functional roles for such factors, as well as for additional factors whose roles in resection and HR still await identification.
