# Supplementary material for: A High-Throughput, Flow Cytometry-Based Method to Quantify DNA-End Resection in Mammalian Cells
Source: Cytometry A. 2012 Aug 14;81A(10):922–8. doi: 10.1002/cyto.a.22155 (PMC3601416; doi:10.1002/cyto.a.22155)
Supplement: Supplementary file 3 [file cyto0A81-0922-SD3.doc]

**Supplementary Information**

**A high-throughput, flow cytometry-based method to quantify DNA-end resection in mammalian cells**

Josep V. Forment, Rachael V. Walker, and Stephen P. Jackson

Fluorescent Reagent Description

| **Characteristic being measured** | **Analyte** | **Analyte Detector** | **Reporter** | **Manufacturer** | **Dilution** | **Clone** | **Catalogue Number** |
| --- | --- | --- | --- | --- | --- | --- | --- |
| Single- stranded DNA | RPA2 | Mouse anti-RPA32  (RPA2 Ab#1) | Primary antibody | Merck | 1:100 | RPA34-20 | NA19L |
| Single-stranded DNA | RPA2 | Mouse anti- RPA32  (RPA Ab #2) | Primary antibody | Abcam | 1:200 | RPA2 9HB | ab2175 |
| Mouse anti-RPA2 antibody | Secondary antibody | Goat anti-mouse | Alexa Fluor 488 | Molecular Probes | 1:200 | N/A | A11029 |
| Damaged DNA | Histone variant H2A.X phosphorylated on Ser-139 | Rabbit anti-γH2AX | Primary Antibody | Cell Signalling | 1:100 | N/A | 2577 |
| Rabbit anti-γH2AX antibody | Secondary Antibody | Goat anti-rabbit | Alexa Fluor 647 | Molecular Probes | 1:200 | N/A | A21245 |
| DNA content | DNA in all cells | DAPI | DAPI | Sigma | 1 µg/ml | N/A | D9542 |
| Cells actively cycling | Replicated DNA | Click-iT EdU | Alexa Fluor 647 | Life Technologies | 10 µM | N/A | C10419 |

Configuration of Beckman Coulter CyAn ADP

| **Laser** | **Power** | **Laser Manufacturer** | **Installation Date** |
| --- | --- | --- | --- |
| 488 nm Solid state | 20 mW | Coherent | 2007 |
| 635 nm Solid state | 25 mW | Beckman Coulter | 2007 |
| 405 nm Solid state | 25 mW | Coherent | 2007 |

Acquisition settings = no custom alterations

| **Reporter** | **Log/Lin scale** | **Channel** | **Laser excitation** | **Emission Filter** | **Installation Date** | **Voltage** |
| --- | --- | --- | --- | --- | --- | --- |
| Alexa 647 – γH2AX | Log | FL8 -APC | 633 nm | 670/30 | 2007 | 450 |
| Alexa 647 – EdU | Log | FL8 - APC | 635 nm | 670/30 | 2007 | 600 |
| DAPI | Lin (area and height) | FL6 – Violet 1 | 405 nm | 450/50 | 2007 | 425 |
| Alexa 488 – RPA2 | Log | FL1 - FITC | 488 nm | 530/30 | 2007 | 500 |

**Supplementary Figure legends**

**Figure S1:** Gating scheme for total and CPT-induced RPA staining. Blue squares represent the gates used in each panel to select events for further analysis. **(A)** Forward versus Side Scatter plot to eliminate debris. **(B)** Gating single cells with pulse height versus pulse area of the DAPI channel. **(C)** Alexa Fluor 488 versus DAPI to gate the total amount of RPA positive cells (same samples as in Fig. 2A). **(D)** Intensity of the total RPA signal (as shown in Fig. 2B, middle panel) was calculated using the geometrical mean of the Alexa Fluor 488 signal in the gated populations from (C). **(E)** CPT-induced RPA-positive cells (right) are gated using the highest RPA intensity signal in non-treated cells (left; quantification in Fig. 2B, right panel). Note that the samples are the same shown in (C). 30 000 events were counted.

**Figure S2:** Alternative measurement of the differences in the intensity of RPA2 signals in cells left untreated or treated with CPT. The gating scheme prior to the analysis shown in this figure is the same used in Fig. S1A-B. **(A)** Alexa Fluor 488 versus DAPI also allows gating of the mid-S phase population. Since the RPA2 signal in this population follows a typical log-normal distribution in both conditions, it can also be used to quantify. **(B)** Intensity of the total RPA2 signal was calculated using the geometrical mean of the Alexa Fluor 488 signal in the gated populations from (A).
