# Supplementary material for: A High-Throughput, Flow Cytometry-Based Method to Quantify DNA-End Resection in Mammalian Cells
Source: Cytometry A. 2012 Aug 14;81A(10):922–8. doi: 10.1002/cyto.a.22155 (PMC3601416; doi:10.1002/cyto.a.22155)
Supplement: Supplementary file 4 [file cyto0A81-0922-SD4.doc]

**Cytometry Part A**

**Author Checklist: Location of MIFlowCyt-Compliant Items**

| **Requirement** | **Requested Information or Specific Location in Manuscript (Please provide chapter/section titles and areas instead of page numbers)** |
| --- | --- |
|  |  |
| 1.1. Purpose | Abstract, Introduction (last paragraph) |
| 1.2. Keywords | Abstract |
| 1.3. Experiment variables | Materials and methods, Results |
| 1.4. Organization name and address | Title page |
| 1.5. Primary contact name and email address | Title page |
| 1.6. Date or time period of experiment | Materials and methods |
| 1.7. Conclusions | Discussion |
| 1.8. Quality control measures | Material and methods, Results, Supplementary material |
| 2.1.1.1. (2.1.2.1., 2.1.3.1.) Sample description | Materials and methods |
| 2.1.1.2. Biological sample source description | Materials and methods |
| 2.1.1.3. Biological sample source organism description | N/A |
| 2.1.2.2. Environmental sample location | N/A |
| 2.3. Sample treatment description | Materials and methods, Figure legends |
| 2.4. Fluorescence reagent(s) description | Materials and methods, Supplementary material |
| 3.1. Instrument manufacturer | Materials and methods, Supplementary material |
| 3.2. Instrument model | Materials and methods, Supplementary material |
| 3.3. Instrument configuration and settings | Materials and methods, Supplementary material |
| 4.1. List-mode data files | Flow Repository (www.flowrepository.org) with ID FR-FCM-ZZYT^*^ |
| 4.2. Compensation description | Materials and methods |
| 4.3. Data transformation details | Materials and methods, Supplementary material |
| 4.4.1. Gate description | Materials and methods, Supplementary material |
| 4.4.2. Gate statistics | Materials and methods, Figures, Supplementary material |
| 4.4.3. Gate boundaries | Materials and methods, Results, Supplementary material |

^*^*http://flowrepository.org/id/RvFrqqbA3YksIr8eWBmvf9P3XVBzaGFsILDkmG78Ii3MrsKs9EQ5b85F5f610Yig*

**1.1. Purpose**

Replication protein A (RPA) is an essential trimeric protein complex that binds to single-stranded DNA (ssDNA) in eukaryotic cells and is involved in various aspects of cellular DNA metabolism, including replication and repair. Although RPA is ubiquitously expressed throughout the cell cycle, it localizes to DNA replication forks during S phase, and is recruited to sites of DNA damage when regions of ssDNA are exposed. During DNA double-strand break (DSB) repair by homologous recombination (HR), RPA recruitment to DNA damage sites depends on a process termed DNA-end resection. Consequently, RPA recruitment to sub-nuclear regions bearing DSBs has been used as readout for resection and for ongoing HR. To complement assays that assess HR by measuring its end points (10-13), and as a rapid means of detecting proteins involved in HR-mediated repair, many researchers have used microscopic detection of RPA or other HR-associated proteins within punctate sub-nuclear foci at DNA damage sites to assess progression into and through the various stages of HR (14-18). Although defects in RPA-focus formation as measured by these methods can be clear when core resection proteins are mutated or absent, recent research has highlighted the fact that many proteins play a role in this process (19), with defects in some of them leading to often quite subtle phenotypes that can be difficult to quantify by standard microscopy-based assays. As described below, to help circumvent such shortcomings, we have developed a flow-cytometry method to quantitatively analyze RPA accumulation as readout for DNA-end resection, a method that should find utility in various studies analyzing HR and associated events and in defining how various proteins influence such processes.

**1.2. Keywords**

Flow cytometry, replication protein A, DNA damage, DNA replication, DNA repair, homologous recombination, DNA-end resection.

**1.3. Experiment variables**

RPA staining to measure cell proliferation and DNA-damage repair by HR.

**1.4. Organization name and address**

The Gurdon Institute and Department of Biochemistry, University of Cambridge. Tennis Court Road, CB2 1QN, Cambridge, UK.

Cambridge Stem Cell Institute, Wellcome Trust Centre for Stem Cell Research, University of Cambridge. Tennis Court Road, CB2 1QR , Cambridge, UK.

**1.5. Primary contact name and email address**

Correspondence to J.V. Forment: j.forment@gurdon.cam.ac.uk or S.P. Jackson: s.jackson@gurdon.cam.ac.uk

**1.6. Date or time period of experiment**

All experiments were carried out between September 2011 and March 2012.

**1.7. Conclusions**

We have developed a flow-cytometry based assay that uses anti-RPA antibodies to detect RPA-ssDNA formation in cells. By extracting non DNA-bound RPA before sample fixation, we were able to distinguish non-replicating from replicating cells, thus establishing RPA as a new marker for cellular proliferation that can be employed in flow cytometry studies. We have also shown that this assay can identify increased RPA signals caused by DNA-damaging agents. The fact that the assay can detect significant differences in signals after DNA damage in both the percentage of cells showing RPA staining and the intensity of RPA staining in a given cell population opens the possibility of its use as a more quantitative and unbiased way to measure RPA-coated ssDNA formation in various experimental settings.

**1.8. Quality control measures**

For the use of RPA as a marker of DNA replication: co-staining of RPA with EdU. To confirm that the increased RPA signal observed after camptothecin treatment was due to DNA damage: co-staining of RPA with  γH2AX. To confirm that the increased RPA signal observed after DNA damage was a consequence of DNA-end resection: disappearance of RPA signals in cells depleted of the nuclease CtIP.

**2.1.1.1. (2.1.2.1., 2.1.3.1.) Sample description**

Human osteosarcoma U2OS cells (ATCC #HTB-96) were used throughout and were grown in Dulbecco’s modified Eagle medium supplemented with 10% fetal bovine serum, glutamine and antibiotics. Camptothecin and etoposide were from Sigma. Transfection with small-interfering RNAs (siRNAs) was performed by using Lipofectamine RNAiMAX (Invitrogen) following the manufacturer’s instructions.

**2.1.1.2. Biological sample source description**

Human osteosarcoma U2OS cells (ATCC #HTB-96)

**2.3. Sample treatment description**

Samples were collected from 6 cm dishes, with cells being 60-90% confluent (~0.5-1 x 10^6^ cells). If EdU detection was performed, cells were pulse-labeled with 10 μM EdU for 30 min before collection. Cells were treated with 1 μM of camptothecin (CPT) for 1 h or 5 μM ETP for 4 h before harvesting.

**2.4. Fluorescence reagent(s) description**

Secondary antibodies: goat anti-mouse Alexa Fluor 488 (Molecular probes, 1:200 dilution), goat anti-rabbit Alexa Fluor 647 (Molecular probes, 1:200 dilution). 5-ethynyl-2'-deoxyuridine (EdU) incorporation was measured by using the Click-iT EdU Alexa Fluor 647 Flow cytometry kit (Life Technologies) following manufacturer’s instructions.

| **Characteristic being measured** | **Analyte** | **Analyte Detector** | **Reporter** | **Manufacturer** | **Dilution** | **Clone** | **Catalogue Number** |
| --- | --- | --- | --- | --- | --- | --- | --- |
| Single- stranded DNA | RPA2 | Mouse anti-RPA32  (RPA2 Ab#1) | Primary antibody | Merck | 1:100 | RPA34-20 | NA19L |
| Single-stranded DNA | RPA2 | Mouse anti- RPA32  (RPA Ab #2) | Primary antibody | Abcam | 1:200 | RPA2 9HB | ab2175 |
| Mouse anti-RPA2 antibody | Secondary antibody | Goat anti-mouse | Alexa Fluor 488 | Molecular Probes | 1:200 | N/A | A11029 |
| Damaged DNA | Histone variant H2A.X phosphorylated on Ser-139 | Rabbit anti-γH2AX | Primary Antibody | Cell Signalling | 1:100 | N/A | 2577 |
| Rabbit anti-γH2AX antibody | Secondary Antibody | Goat anti-rabbit | Alexa Fluor 647 | Molecular Probes | 1:200 | N/A | A21245 |
| DNA content | DNA in all cells | DAPI | DAPI | Sigma | 1 µg/ml | N/A | D9542 |
| Cells actively cycling | Replicated DNA | Click-iT EdU | Alexa Fluor 647 | Life Technologies | 10 µM | N/A | C10419 |

**3.1 and 3.2. Instrument manufacturer and model**

Samples were analyzed by using a Beckman Coulter CyAn ADP Flow Cytometer.

**3.3. Instrument configuration and settings**

DAPI was excited with a 405 nm laser and emissions collected via a 450/50 filter. The Alexa 488 fluorochrome was excited by a 488 nm laser, and the emitted light collected via a 530/40 filter. A 635 nm laser was used for the Alexa 647 fluorochrome and the emission collected via a 670/30 filter.

| **Laser** | **Power** | **Laser Manufacturer** | **Installation Date** |
| --- | --- | --- | --- |
| 488 nm Solid state | 20 mW | Coherent | 2007 |
| 635 nm Solid state | 25 mW | Beckman Coulter | 2007 |
| 405 nm Solid state | 25 mW | Coherent | 2007 |

| **Reporter** | **Log/Lin scale** | **Channel** | **Laser excitation** | **Emission Filter** | **Installation Date** | **Voltage** |
| --- | --- | --- | --- | --- | --- | --- |
| Alexa 647 – γH2AX | Log | FL8 -APC | 633 nm | 670/30 | 2007 | 450 |
| Alexa 647 – EdU | Log | FL8 - APC | 635 nm | 670/30 | 2007 | 600 |
| DAPI | Lin (area and height) | FL6 – Violet 1 | 405 nm | 450/50 | 2007 | 425 |
| Alexa 488 – RPA2 | Log | FL1 - FITC | 488 nm | 530/30 | 2007 | 500 |

**4.1. List-mode data files**

<http://flowrepository.org/id/RvFrqqbA3YksIr8eWBmvf9P3XVBzaGFsILDkmG78Ii3MrsKs9EQ5b85F5f610Yig>

**4.2. Compensation description**

As each of the fluorochromes was excited by a different laser no compensation was necessary.

**4.3. Data transformation details**

Cells were gated on the Forward versus Side Scatter plot to eliminate debris, and then single cells were gated by using a dot-plot showing the pulse height versus pulse area of the DAPI channel (see Supp. Fig. S1). Post-acquisition analysis was performed with FlowJo software (Tree Star).

**4.4.1. Gate description**


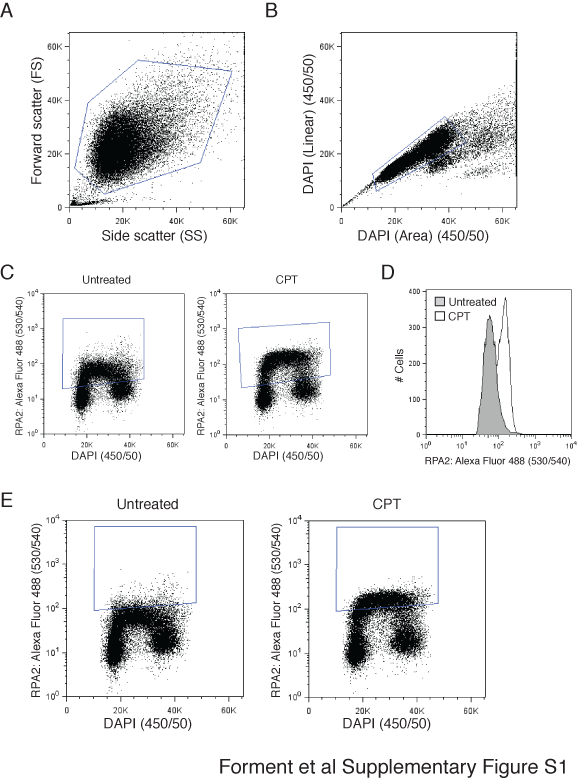


**Figure S1:** Gating scheme for total and CPT-induced RPA staining. Blue squares represent the gates used in each panel to select events for further analysis. **(A)** Forward versus Side Scatter plot to eliminate debris. **(B)** Gating single cells with pulse height versus pulse area of the DAPI channel. **(C)** Alexa Fluor 488 versus DAPI to gate the total amount of RPA positive cells (same samples as in Fig. 2A). **(D)** Intensity of the total RPA signal (as shown in Fig. 2B, middle panel) was calculated using the geometrical mean of the Alexa Fluor 488 signal in the gated populations from (C). **(E)** CPT-induced RPA-positive cells (right) are gated using the highest RPA intensity signal in non-treated cells (left; quantification in Fig. 2B, right panel). Note that the samples are the same shown in (C). 30 000 events were counted.

**4.4.2. Gate statistics**


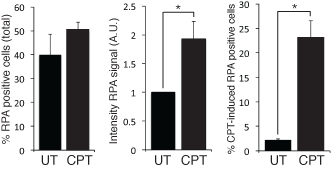


Quantification of total amounts of RPA2-positive cells (left panel), the intensities of RPA2 signals (normalized to the intensity in untreated samples; middle panel), and the total amounts of DNA-damage induced RPA-positive cells (using the population gated in (A); right panel). For an example of the gating scheme, see Supplementary Figure S1. Results are averages of at least three independent experiments and the error bars correspond to standard deviations. (*) Denotes statistically significant differences (*P* value < 0.05).


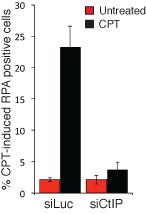


Quantifications (right panel) were performed with the same gating scheme as in Fig. 2B (see Supp. Fig. S1). Results are averages of at least three independent experiments and the error bars correspond to standard deviations.

**4.4.3. Gate boundaries**

To more clearly reflect the differences in RPA2 staining between untreated and treated cells, we defined a gate at the higher intensity level of RPA staining for most cells (> 95%) in untreated conditions (dashed square in Fig. 2A; see Supp. Fig. S1) and used this as the basis for further quantifications.


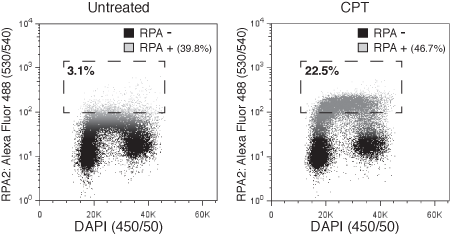


DNA damage increases the intensity of RPA2 signals. Cells were treated with 1 μM of camptothecin (CPT) for 1 h before harvesting. The dashed square marks the gate (showing the percentage of cells in it) used for quantification in the right panel in (B).
